# Collagen-Based Biomimetic Systems to Study the Biophysical Tumour Microenvironment

**DOI:** 10.3390/cancers14235939

**Published:** 2022-11-30

**Authors:** Alessandra Cambi, Maurizio Ventre

**Affiliations:** 1 Department of Cell Biology, Radboud University Medical Center, Geert Grooteplein Zuid 26-28, 6525 GA Nijmegen, The Netherlands; 2 Department of Chemical, Materials and Industrial Production Engineering, University of Naples Federico II, 80125 Naples, Italy; 3 Interdisciplinary Research Centre on Biomaterials, University of Naples Federico II, 80125 Naples, Italy; 4 Center for Advanced Biomaterials for Healthcare@CRIB, Fondazione Istituto Italiano di Tecnologia, 80125 Naples, Italy

**Keywords:** mechanobiology, collagen, extracellular matrix, stiffness, cell cultures, gel, cell derived matrices, decellularized tissues

## Abstract

**Simple Summary:**

It is increasingly acknowledged that cells in the body not only sense biochemical signals, but also feel and respond to mechanical signals. Tissues and organs, for example, have very different stiffnesses and can be very stiff like bone or soft like lungs or the gut. This difference in stiffness is mostly due to the presence of collagen, a protein present in between cells that provides structural support to organs and tissues. Together with proteins such as fibronectin, collagen forms the so-called extracellular matrix. Excessive presence or defective organization of collagen in this matrix alter the normal mechanical properties of organs and tissues and accompany diseases such as fibrosis and cancer. This review describes the systems used in the laboratory to mimic this matrix and investigate how cells respond to different mechanical forces in health and disease.

**Abstract:**

The extracellular matrix (ECM) is a pericellular network of proteins and other molecules that provides mechanical support to organs and tissues. ECM biophysical properties such as topography, elasticity and porosity strongly influence cell proliferation, differentiation and migration. The cell’s perception of the biophysical microenvironment (mechanosensing) leads to altered gene expression or contractility status (mechanotransduction). Mechanosensing and mechanotransduction have profound implications in both tissue homeostasis and cancer. Many solid tumours are surrounded by a dense and aberrant ECM that disturbs normal cell functions and makes certain areas of the tumour inaccessible to therapeutic drugs. Understanding the cell-ECM interplay may therefore lead to novel and more effective therapies. Controllable and reproducible cell culturing systems mimicking the ECM enable detailed investigation of mechanosensing and mechanotransduction pathways. Here, we discuss ECM biomimetic systems. Mainly focusing on collagen, we compare and contrast structural and molecular complexity as well as biophysical properties of simple 2D substrates, 3D fibrillar collagen gels, cell-derived matrices and complex decellularized organs. Finally, we emphasize how the integration of advanced methodologies and computational methods with collagen-based biomimetics will improve the design of novel therapies aimed at targeting the biophysical and mechanical features of the tumour ECM to increase therapy efficacy.

## 1. Introduction

Biological tissues are material objects. Depending on the anatomical location, tissues are characterized by complex structures and highly specific compositions. The architects of such an extraordinary variety of shapes and functions are the cells. These lay down and assemble the extracellular matrix (ECM), which in turn contains signals that guide and sustain cells in a sort of dynamic reciprocity that ultimately defines the homeostasis of the tissue. While we are familiar with biochemical signals (growth factors, hormones) that interact with cells through specific receptors, we must also acknowledge that biophysical signals like ECM topography, elasticity and porosity are as powerful as the biochemical signals in eliciting cell responses that include proliferation, apoptosis, differentiation and migration. In particular, cells perceive the biophysical microenvironment through specialized receptors. Although several collagen receptors are known, we will here focus on integrins. These cluster together to form mechanically stable pegs, termed focal adhesions (FAs), over which the cytoskeleton assembles. Several proteins constituting the FAs are signalling molecules with a mechanosensitive behaviour, i.e., when cytoskeleton-generated forces of sufficient magnitude act on them, these molecules may change their conformation and/or activity, triggering signalling pathways [1,2]. Also, the cytoskeletal contractility itself may alter the activity of several cytoplasmic molecules with signalling functions, and the cytoskeleton components variously interact with transcription factors, ultimately affecting their nuclear import or activity [3,4,5,6]. Finally, the cytoskeleton is variously connected to the nuclear envelope and differently structured cytoskeleton assemblies alter nuclear shape, chromosome positioning and chromatin condensations state [7,8]. These pieces of evidence depict a scenario in which adhesions, the cytoskeleton and the nucleus constitute a unique tripartite module, in which modulating one element inevitably affects the other two and altogether they will eventually regulate gene transcription and expression. This tripartite module is the fundamental basis of mechanobiology, for which cells perceive mechanical forces (mechanosensing) and transduce them (mechanotransduction) into biochemical events. Mechanobiology is not limited to cell-generated mechanical forces. In fact, as the ECM is continuously connected to FAs, exogenous forces can be transmitted to the cytoskeleton by passing through the ECM fibres and the FAs. The way cells perceive the biophysical microenvironment and exert contractile force, as well as the way forces are distributed through the ECM, have profound implications not only in the morphogenesis and maintenance of tissues, but also in establishing the onset and progression of diseases. Aberrant ECMs have been observed in a variety of diseases including osteoarthritis, cirrhosis, organ fibrosis and cancer, and altered mechano- sensing or -transduction capabilities have been related to the development of muscular dystrophies, cardiomyopathies and cancer [9,10,11,12].

The intricate relationships connecting the ECM biophysical features with gene expression have been only partially disclosed, and although some molecular players involved in mechanotransduction have been identified, our ability to control them, either in vitro or in vivo is still modest. This calls for the development of techniques and devices to deconstruct the mechanosensing and mechanotransduction processes, thus providing elements to define a unifying model that connects the mechanical and molecular regulatory aspects of cell behaviour. The ideal platform to unravel these events would be an “artificial” ECM, in which the biochemical and biophysical characteristics affecting the cell adhesion processes could be freely modulated in an orthogonal manner. Real ECMs are a very complex environment constituted by a myriad of macromolecular components, whose structural assembly goes well-beyond any conceivable man-made structure. Attempts in engineering ECM mimics in vitro resulted in the development of simplified, yet versatile, systems such as cell responsive polyethylene glycol (PEG)-based hydrogels [13], hydrogels with tuneable stiffness [14] and peptide-base self-assembling gels [15,16].

Different approaches encompass the use of natural ECMs (either as a whole or part of them) suitably modified to study mechanobiology-related problems, or controlling and conditioning cells to obtain specific responses. Recently, there has been a renewed interest towards native ECMs as cell culturing platforms either as purified protein extracts, cell derived matrices (CDMs) or as decellularized tissues/organs. A demonstration of this trend is the growing number of tissue- or organ-on-chip devices that use ECM components, that have been developed to study complex biological phenomena or that have been used for drug screening/repurposing applications [17,18,19]. Despite being capable of better mimicking the in vivo context, natural ECMs have clear limitations: the intrinsic biological variability among apparently analogous samples, and the scarce possibilities to alter their mechanical, structural and compositional properties challenge the wide-spread use of EMC-derived matrices in laboratory.

Here, we discuss an array of biomimetic culturing systems derived from ECM macromolecular components. Emphasis will be given to collagen-based devices, owing to the importance of this protein in ECMs. The culturing systems will be presented in an order of increasing structural and molecular complexity, spanning from simple 2D substrate to complex decellularized organs. Finally, we will highlight computational models as important tools to describe and predict cell response to ECM physicochemical features that could eventually be tuned to elicit specific cellular responses.

## 2. The Importance of Collagen in the ECM

**Figure 1:**
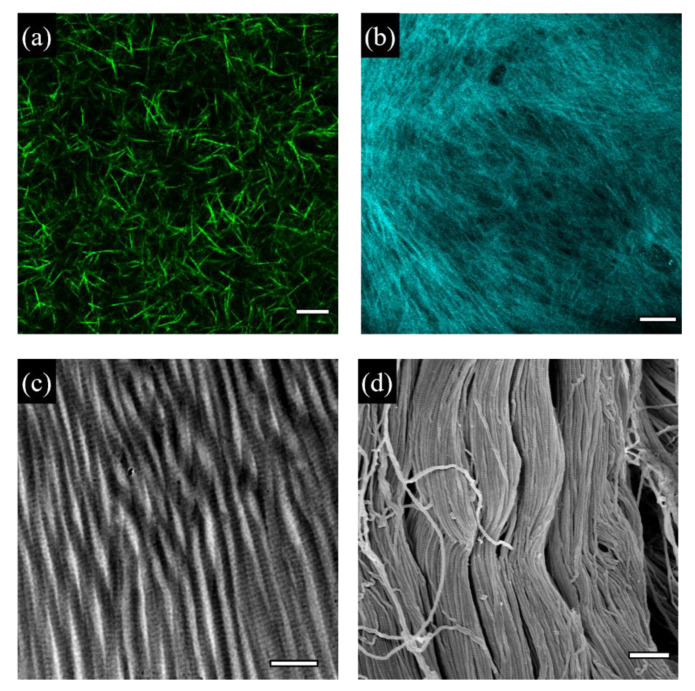
Examples of collagen-based materials for cell cultures. (**a**) Reconstituted bovine collagen type I gel (2.4 mg/mL) observed with confocal microscopy. Bar 10 μm. (**b**) MC3T3 cell-derived matrix, collagen fibrils observed in confocal second harmonic generation imaging. Bar 50 μm. Collagen fibrils in a decellularized ovine skin sample observed with TEM (**c**) or SEM (**d**). Bars 400 nm in (**c**) or 1 μm in (**d**).

The most abundant and probably most studied ECM constituents are collagens [20]. In vertebrate connective tissues, collagens act as a fundamental load-bearing scaffold. Also, they are endowed with important bioactive properties, and, unlike the vast majority of synthetic polymers, they can spontaneously self-assemble to form fibrils, membranes or gels (Figure 1).

To date, 28 different types of collagens have been found [21], all sharing the common feature that the elementary molecule is constituted by a triple helix, which can then spiralize to form fibres. Collagen fibres follow a hierarchical assembly of subunits, such as microfibrils, sub-fibrils and finally triple helices. Fibril-forming collagens (i.e., types I, II, III, V and XI) are the supporting framework of the tissues. The mixture of different collagen types may interact to regulate certain physicochemical properties of collagen fibres. For instance, Type I, III and V collagens are usually found together in the same fibres, and their relative amount is thought to regulate fibre size and mechanical properties [22,23,24]. Other molecules such as glycosaminoglycans (GAGs) can assemble onto the fibres to further modify the functions, biological activity or mechanical properties of the fibrous network [25,26,27].

In normal and healthy tissues, ECM is constantly remodelled by cells to preserve tissue structural integrity and functions. This occurs thanks to a delicate balance between biomolecule synthesis, assembly, degradation and removal. In the case of collagens, new molecules are produced and delivered to the extracellular space to replace old ones. Proteolytic enzymes (matrix metalloproteinases, MMPs) and inhibitors thereof, are expressed in a tightly controlled manner to selectively degrade the matrix. Other enzymes that alter the chemical/physical features of the biomolecules, such as the crosslinking enzyme lysyl oxidase (LOX, which covalently cross-links collagen fibrils), participate in the remodelling process. Deviations from this equilibrium not only impair tissue functionality, but may also promote and sustain the progression of complex pathologies. In tumours, collagen remodelling is dysregulated: an excess in collagen production accumulates in the stroma surrounding the tumour mass in a process also referred to as desmoplasia [28,29]. Here, collagens are heavily crosslinked and form straight fibres that together cause a local stiffening of the microenvironment. Such an alteration of the tissue mechanics, feedback signals to the residing cells. This alters their proliferation, differentiation as well as migration and can negatively affect the efficacy of anti-tumour therapies by reducing drug penetration or by dampening the immunosuppressive activity of tumour-associated immune cells (as reviewed in [30]). In fact, the morpho-physical features of the tumour stroma are used as a prognostic marker of the tumour progression, as an increased collagen density and the level of collagen alignment correlate with a poor prognosis of several tumour types [31,32,33,34].

Considering the importance of collagens in both physiological and pathological conditions, collagen-based cell culturing systems have been developed in the past decades to address important questions in mechanobiology research and, in particular, on cancer mechanobiology. From an engineering perspective, collagen possesses several features that provide sufficient versatility for the fabrication of different cell-culturing devices. Collagen molecules can be extracted from natural tissues, or cells can be stimulated to secrete and assemble rudimentary collagen-rich ECMs in vitro. The extracted collagen molecules retain the ability to self-assemble in vitro to form fibrils, which can be grown on surfaces or may form weak 3D gels. As type I is the most abundant collagen type, the majority of in vitro systems are based on this type of collagen. In the remainder of this review we will mostly refer to type I collagen-based systems unless otherwise stated. We must emphasize that, without the intervention of cells, self-assembled collagen fibrils in vitro do not exactly replicate the structures of ECMs in vivo, as collagen concentration, assembly and consequently the mechanical properties are different. However, by carefully modulating the processing conditions, it is possible to replicate in vitro some of the chemical/physical features of the native microenvironment. In this review, we will compare and contrast collagen-based and collagen-functionalized culturing systems, discussing how their chemical-physical characteristics relate to specific applications in the context of the mechanobiology of the tumour microenvironment.

## 3. Collagen Substrates for 2D Cultures

Engineering and fabricating surfaces that are able to finely control the cell adhesive processes has profound implications in the regulation of a wide variety of cell functions that go well beyond cell attachment. For instance, endothelial cells confined on small adhesive areas are prone to apoptosis, whereas proliferation is observed on large areas [35]. Similarly, mesenchymal stem cells (MSCs) cultivated on soft substrates matching the brain stiffness are directed towards neurogenesis, whereas they undergo osteogenesis on stiff gels [36]. Also, promoting MSC elongation with specifically designed topographies induces neuron transdifferentiaiton [37]. These and many other examples emphasize the central role of adhesion-mediated signalling, contractility and cytoskeleton-mediated transcription factor shuttling in the mechanotransduction process. Homogeneous coating of stiff materials (like glass or polystyrene) with protein solutions is scarcely effective for regulating the formation and maturation of FAs, which would result in poor control of the FA signalling activity, unpredictable development of cytoskeletal structures and cell-generated forces. To partly solve this issue, micro and nanopatterning techniques have been developed with the common aim of defining the spatial arrangement of ligands, and hence controlling the geometrical features of Fas, cell shape and cytoskeleton assembly. Some of these techniques proved to be effective in controlling cell adhesion events at receptor level (e.g., block copolymer micelle nanolithography, electron beam lithography), FA level (replica moulding, hot embossing) or at cell level (microcontact printing, photopatterning). For instance, using block copolymer micelle nanolithography, Arnold et al. fabricated quasi- hexagonal arrays of gold nanoparticles on glass substrates, in which particle displayed the RGD ligand surrounded by antiadhesive PEG [38]. The authors found that adhesion formation required an interparticle, i.e., interligand spacing, in the 58–73 nm range, whereas sparser particles disrupted adhesion formation. Similarly, regular square lattices of nanopit arrays (120 nm wide pits with lateral spacing of approximately 300 nm) partially abrogated MSC contractility and induced cells to retain multipotency [39]. Conversely, disordered patterns with small offsets in the lateral spacing promoted the formation of larger Fas, which eventually resulted in increased osteogenesis [40]. Also, linear gratings of 700 nm ridges and grooves aligned Fas and contractile forces of hMSCs, ultimately guiding cell self-organization and tenogenesis [41].

In vivo, different signals influencing cell adhesion, such as ligand types and their spatial positioning, topographic relieves and mechanical properties, are integrated with ECM elements and manifest themselves simultaneously. Concerning collagen fibrils, type I collagen possesses the adhesive peptide motif GFOGER that is evenly spaced at 68 nm in light of the staggered alignment of collagen I molecules in the fibril, a staggering that is also referred to as the D-period [42]. When interacting with collagen fibrils, cells do not only perceive the spatial patterning of the ligand, but also the topography of the fibril and its mechanical property. The successful integration of these aspects into artificial systems would result in material models possessing a comprehensive set of instructions that can, in principle, affect diverse cellular responses including adhesion, migration and differentiation. Although this integration is challenging, in vitro approaches promoting the directional growth of collagen molecules on 2D surfaces have been developed.

**Figure 2:**
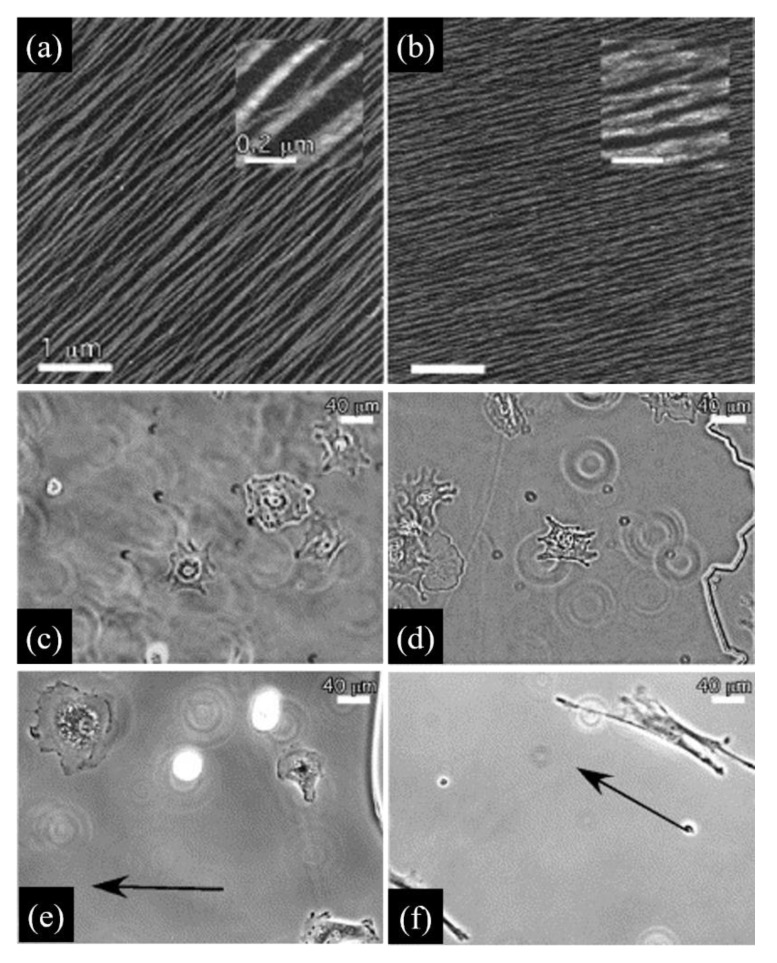
Effect of the architecture of nanostructured type I collagen matrices on fibroblast. (**a**) Aligned collagen fibrils displaying a D-periodic banded pattern obtained using a buffer containing K+ ions; (**b**) collagen fibrils without D-periodicity obtained with a buffer containing Na+ ions. Bars 1.0 μm (inset 0.2 μm). (**c**) Mouse dermal fibroblasts cultivated on mica; (**d**) on unaligned collagen fibrils; (**e**) on aligned collagen fibrils lacking D-periodicity; (**f**) on aligned D-periodic collagen fibrils. Only cells cultivated on (**a**) type substrates display elongated morphology and directional motility along fibril direction (black arrow). Bars 40 μm. Reprinted from Journal of Molecular Biology, 349, Poole et al. Molecular-Scale Topographic Cues Induce the Orientation and Directional Movement of Fibroblasts on Two-Dimensional Collagen Surfaces, 380–386, 2005 with permission form Elsevier, [46]. Permissions regarding further reuse of these figures should be directed to the Elsevier.

Collagen fibrillogenesis is an entropy driven process that is affected by environmental conditions such as temperature, pH, ionic strength and collagen concentration. If not properly directed, random fibril growth and orientation result in weak gels. However, collagen molecules can adsorb on surfaces acting as nucleation points for the fibrillogenesis. Mica surfaces exhibiting a specific crystallographic orientation determine the alignment of collagen fibres and direct their growth [43]. When fibrillogenesis is induced in buffers mimicking the eukaryotic cytoplasmic environment at pH 7.2, collagen fibrils display morphological features similar to those observed in vivo, such as the characteristic D-period and a thickness of 3 nm, corresponding to the average diameter of collagen microfibrils in real ECMs [44,45]. In buffers deprived of potassium, collagen is still able to form parallel and aligned fibrils, but these lacked the banded pattern [44], thus emphasizing the importance of the chemical environment in fibrillogenesis (Figure 2a,b).

The importance of proper fibrillogenesis is demonstrated by the observation that fibroblasts seeded on 2D aligned collagen layers adhere, elongate and migrate along the fibre direction as a direct consequence of the strong contact guidance exerted by the orderly arrangement of fibrils. However, such a directional motility is observed only when the fibrils display the characteristic D-periodicity, as fibroblasts seeded on type I collagen lacking such a periodicity are randomly oriented and exhibit impaired motility [46] (Figure 2c,f). By exploiting anisotropic aligned collagen I fibrils on mica, Kirmse et al. investigated the interdependency of cell-collagen adhesion, cell-generated force transmission and proteolytic remodelling caused by MV3 melanoma cells on collagen [47]. As the experimental model allowed for a direct visualization of fibrillar features and cell compartments, the authors showed that collagen remodelling depended on the functionality of a2b1 integrins, the structure of the actin network and myosin II activity, which are features tightly connected to the mechanical signalling provided by the extracellular environment of aligned fibrils. Random collagen matrices did not induce substantial alteration of the matrix, emphasizing the central role of mechanosensing in matrix remodelling. More recently, Wang et al. used aligned or non-aligned type I collagen fibrils on mica to study the contact guidance behaviour of two invasive breast cancer cell lines (MDA-MB-231 and MTLn3) [48]. These exhibited very different migratory behaviours that were interpreted as a result of the different levels of traction forces exerted by the two cells. In particular, highly contractile MDA-MB-231 exert forces that promote focal adhesion—ollagen fibril coalignment, thus resulting in a directed migration, whereas MTLn3 exerting lower traction forces display a random migration. Hence, the anisotropic mechanical stimulus provided by aligned fibrils is permissive, but not sufficient to drive an effective directed migration.

Despite the interesting biomimetic features integrating adhesive and topographic signals, 2D aligned fibrillar collagen coatings on rigid substrates (such as mica) do not allow further extensive manipulations. To partially solve this issue, Wang et al. transferred the aligned type I collagen fibrils onto polydimethylsiloxane (PDMS) or polyacrylamide (PA) gels, two widely used materials for mechanobiological studies [49]. This approach allowed analysis of the role of elasticity, dictated by the compliant PDMS or PA substrate, on the behaviour of the cells in contact with the coating of fibrillar collagen. The authors studied the adhesion and motility of MDA-MB-231 human mammary carcinoma cells and MTLn3 rat mammary basal adenocarcinoma. Faster migrations were observed on intermediate stiffness values (i.e., on thick 280 kPa PDMS or 2 kPa PA, as opposed to the GPa stiff glass or 0.2 kPa soft PA gels), whereas directionality was dependent on the cell type: MDA-MB-231 cell directionality slightly increased with substrate stiffness, while MTLn3 cells displayed negligible directionality on the highly flexible substrates [49]. Li et al. integrated carbon nanotubes (CNT) in collagen type I fibres growing on mica to obtain stiffer and highly aligned fibrils [50]. The authors studied the behaviour of SKOV3, a mechanosensitive ovarian cancer cell line, in a material model reminiscent of the tumour stroma formed by highly packed and aligned fibres, which are thought to promote cancer progression. The authors successfully obtained stiffer fibrils (0.84 MPa opposed to 0.27 for bare collagen) with a slightly higher D-period (70 nm vs. 68 nm), the latter resulting in an increase in ligand spacing. On the CNT collagen, SKOV3 cells were more elongated and stiffer with respect to cells on control collagen samples without CNT. Also, SKOV3 cells upregulated the genes associated with the epithelial-mesenchymal transition (EMT) much more in CNT collagen rather than in soft featureless collagen.

Despite their limitations, 2D substrates are invaluable platforms to study cell behaviour and response to microenvironmental stimuli or soluble moieties. They are characterized by an inherent simplicity in the fabrication, direct accessibility to various microscopy techniques (brightfield, AFM, fluorescence, confocal) and are compatible with high throughput systems. Exploiting collagen fibrillogenesis to functionalize synthetic surfaces increases the biological relevance of the system, as this enables cells to perceive topographic features and ligand distributions akin to those observed in vivo. However, 2D surfaces force cells to acquire an apical-basal polarity that is not observed in 3D settings in vivo, which motivates the efforts to exploit collagen self-assembly to generate versatile 3D systems in vitro.

## 4. 3D Fibrillar Collagen Gels

Differently from collagen fibril formation and growth on 2D surfaces, fibrillogenesis in 3D results in the formation of highly hydrated and compliant gels. These are most frequently formed from stock solutions of collagen type I purified from sources such as rat tail or bovine tendon/skin and diluted at a concentration in the 1–5 mg/mL range [51]. Gelification occurs under mild conditions that enable direct cell encapsulation within the gel, resulting in a good 3D cell distribution. This, together with the high transparency, make reconstituted collagen gels ideal platforms to directly visualize and study cell behaviour in a 3D biomimetic environment. Also, 3D collagen gels have found many applications in tissue engineering for the regeneration of soft and hard tissues, in drug delivery and in cosmetic applications [52,53]. The biopolymeric network constituting the gel is composed of short and interconnected fibrils, held together by secondary interactions and physical entanglements. The fine 3D meshwork of fibrils displays a pore size that, although dependent on collagen concentration, is within the order of a few micrometres [54].

Cells embedded in a 3D gel are surrounded by a myriad of fibrils, which they can adhere to. While the formation and growth mechanisms of cell adhesions have been widely studied in 2D settings, the dynamics and exact architecture of adhesion formation and maturation in 3D are less understood. Experimental evidence suggests that diverse protein components of 2D adhesions are also found in their 3D counterparts. In particular, integrins, paxillin, vinculin and zyxin have been localized at adhesion sites in 3D environments for various cell types, including endothelial cells and fibroblasts [55,56], although some studies showed that vinculin, paxillin and talin did not form focal aggregates, but were rather diffuse in the cytoplasm [57]. Interestingly, different levels of protein phosphorylation accompany adhesions in 3D as compared to 2D case. For instance, FAK was found to be less phosphorylated in 3D collagen type I gels compared to 2D glass substrates [58]. Despite these differences in protein location and phosphorylation levels, the mechanosensing mechanisms in 2D and 3D are expected to be quite similar, including the establishment of a molecular clutch between integrins and actin filaments that forms adhesions and mediates force transmission [59].

Local ECM stiffness affects the magnitude of the forces transmitted, as compliant matrices cause clutch slipping, whereas stiffer matrices induce clutch tightening and adhesion gripping. Accordingly, enhanced fibrillar bundling increases local stiffness of the collagen gel and this was found to correlate with adhesion maturation and with the fraction of stable adhesions [59]. Collagen gels have been widely used to study cancer cell behaviour in 3D. A seminal work by Paszek et al. reported that increasing collagen type I density, and hence, stiffness (from 0.17 to 1.2 kPa), alters mammary epithelial cell morphology and disrupts basal polarity. Also, increased gel stiffness promotes FA formation, increase in cytoskeletal tension and the activation of signalling pathways that foster malignant transformation [60].

Both local architecture of the gel and fibril stiffness can dramatically affect adhesion-mediated signalling. Owing to the microscale heterogeneity of the collagen gel, cells may simultaneously interact with fibrils with different mechanical properties, thus forming FAs with different lengths, stabilities and even composition [61]. This raises the important question on what aspect of the mechanical property of a biopolymeric 3D matrix a cell perceives. Differently from a 2D substrate, low bulk elastic moduli of 3D collagen networks do not impair the formation of long FA [61]. At the micrometric scale, however, the local mechanical response is non-homogeneous, i.e., position dependent, and anisotropic, whereas the bulk properties are homogeneous and isotropic. Collagen fibrils have an elastic modulus of several hundreds of MPa in the direction of the fibres [62], whereas they promptly bend or buckle when loaded in different directions, eliciting a very different mechanical response in the cells [63]. Macroscopic mechanical stretching results in a reorientation of collagen fibrils within the gels. Highly aligned collagen fibrils may form from uniaxial stretching. Fibril alignment proved to enhance the efficiency of migration of MDA-MB-231 cancer cells encapsulated in a collagen type I gel by increasing the directional persistence and restricting protrusions along aligned fibres [64].

**Figure 3:**
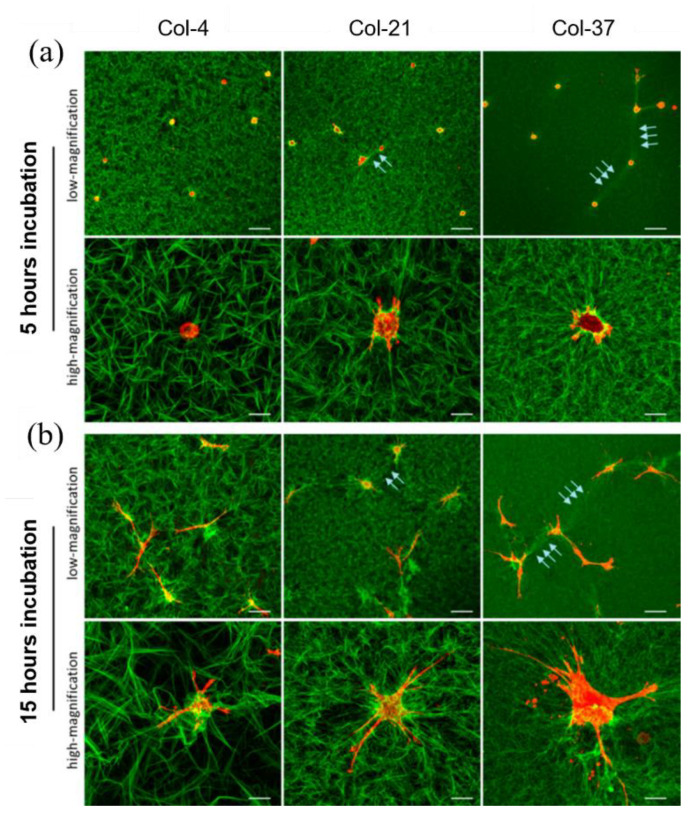
Cell mediated remodelling of fibrillar collagen, gelified at different temperatures. Gels of rat tail collagen type I (3 mg/mL) formed at 4 °C (Col-4) display a loose structure with long and thick collagen fibrils, whereas higher gelification temperatures, i.e., 21 °C and 37 °C (Col-21 and Col-37, respectively) result in gels with a more compact structure and thinner fibres. (**a**) At 5 h after seeding, human MSCs in Col-21 and Col-37 gels start deforming the network by locally aligning and densifying collagen fibrils (green) along actin-rich protrusions (red). No remodelling activity is observed in Col-4 gels. (**b**) At 15 h after seeding, extensive collagen remodelling is observed in Col-21 and Col-37 gels. Network deformation produces collagen lines bridging neighbouring cells (white arrows), in which fibrils are more densely packed and coaligned. Collagen remodelling also occurs on Col-4 gels, although to a lesser extent. Bars, 200 μm (low-magnification panels) or 20 μm (high-magnification panels). Reproduced with permission from Xie et al., ACS Appl. Mater. Interfaces; published by American Chemical Society, 2017, [65]. Permissions regarding further reuse of this figure should be directed to the American Chemical Society.

An additional challenge in creating 3D collagen gels with controlled mechanical properties is represented by the fact that the local architecture and assembly of the fibrillar gel are not fixed: cells that are cultured in these gels will over time exert sufficiently high forces to pull and reorient fibrils (Figure 3). The extent of such a remodelling action depends on the mechanical stability of the network. Compliant fibrils or fibrils pulled in directions different from their principal axis buckle and move towards the cell body. Frequently, collagen densification and fibre coalignment with cell protrusions are indeed observed around the cell body, suggesting an extensive network remodelling [65]. Densification of the matrix and alignment result in an increased local stiffness around the cell that promotes more effective mechanical feedback.

If the cell density is sufficiently high, the local compaction of the network manifests itself as a macroscopic contraction of the gel [66]. Gel contraction has been exploited to induce fibril alignment in a specific direction. Constraining the opposite edges of a gel (usually with yarns or porous plugs) causes an anisotropic compaction and alignment of the gel that ultimately leads to cells reorienting along the direction of highest stiffness. Guiding gel compaction with external constraints has been used to create tissue analogues in vitro, such as tendons (with a uniaxial fibril alignment), blood vessels (circumferential fibril orientation) and heart valve leaflets (commissural alignment) [67,68,69].

In addition to cell alignment, 3D collagen networks can be used to study cell motility under different biophysical conditions. Cells may adopt different migratory strategies, with mesenchymal and amoeboid migration being among the most widely studied. Mesenchymal migration is characterized by FA-dependent motility with the expression of MMP that facilitates cell translocation by cleaving fibrils. Here, Rac1 activation at the leading edge and inhibition of RhoA results in cells migrating with an elongated and polarized morphology. For completeness, it should be noted that Rac1-independent, RhoA-dependent pseudopodial processes and invasive migration have also been reported for cancer cells [70]. Instead, amoeboid migration is dependent on cell contractility and occurs through squeezing movements that enable cells to overcome a physical barrier. Many cells can switch between the two modes depending on the matrix features, including its stiffness [71,72]. Several approaches have therefore been developed to alter micro- and macro-mechanical properties of collagen gels, to eventually modulate the mechanical inputs to and migratory behaviour of cells. Along this line, collagen I–III blends exhibiting different mechanical and architectural features were used to investigate the impact of the mechano-physical signal of the 3D microenvironment on the migration and invasiveness of MDA-MB-231 cancer cells with or without anti-metastasis drugs [73]. It was reported that cytoskeletal contractility-targeting drugs reduced migration speed in sparse gels, whereas migration in dense gels was retarded effectively by inhibiting proteolysis.

Plastic compression is a convenient method to extract water from the gel, thus increasing collagen concentration and gel stiffness, while also reducing pore size and permeability [74,75,76]. To avoid these simultaneous changes and only target the stiffness and flexibility of individual fibrils, methods based on chemical modifications have also been developed. Chemical crosslinking of fibrils by glutaraldehyde or genipin increases the overall stability of the gel, and also reduces degradability [77]. The increase in stiffness produced by the chemical agent depends on the type of compound and its concentration. Treatments may lead to one order of magnitude higher elastic modulus of the gels [78]. As compared to chemical cross-linkers, the use of the LOX enzyme, either directly or through genetically engineered cells, better recapitulates the crosslinking mechanism occurring in vivo during tissue maturation [79,80]. Collagen glycation also proved to be an effective stiffening method, although it is time consuming [81,82]. Finally, the use of ultraviolet crosslinking in the presence of riboflavin possesses the advantage of being a simple and effective method that may also act on a local scale: by using light masks that spatially filter light transmission, it is possible to create patterns or gradients of crosslinked fibrils in the gel [83,84]. Light irradiation, especially in the UV range, may induce cytotoxic effects. Care should be taken when crosslinking has to be performed in the presence of cells. The presence of antioxidants or the use of crosslinkers reacting at longer wavelengths may aid in preventing cell damage.

3D collagen hydrogels certainly represent more physiologically relevant models if compared to 2D collagen coated substrates, as they show key aspects of the supramolecular organization of native tissues. However, they are still far from being ideal systems. Being constituted by a single component, they fail in replicating the heterogeneous composition of native ECMs [85]. Also, the manipulation and modulation of one of their chemical-physical properties inevitably impacts the others (e.g., a change in density leads to a change in stiffness), and achieving independent control of gel properties requires the development of ad hoc strategies [86]. Synthetic hydrogels, such as PA and PEG, can be variously functionalized with different types of moieties such as ligands, sugars, cleavable peptides and growth factors, but their microstructure rarely displays the supramolecular complexity of natural tissues. These limitations prompted the development of cell-generated/derived matrices, either of native tissues/organs or in vitro origin.

## 5. Cell Derived Matrices

Cell-derived matrices (CDMs) gained popularity as semiphysiological biomimetic cell culturing platforms, as they are synthesized and assembled by cells (mainly fibroblasts, osteoblasts and chondrocytes) cultured in vitro. A variety of macromolecular components are found in CDMs, including collagens, fibronectin, GAGs and growth factors. Decellularization is then performed to remove cell membranes, cytoplasmic components and nuclear matter, thus producing a material template that can host different cells. Decellularized CDMs (dCDMs) find applications in regenerative medicine as coatings or parts of scaffolds, as support for cell growth or as models to study cell behaviour in a histologically relevant environment. The biochemical and biophysical characteristics of the dCDMs depend on three factors: the type and density of cells employed, the culturing process and the decellularization process.

The cell source can be constituted by either primary cells or cell lines. Primary cells preserve their native phenotype and are likely to synthesize better mimics of the tissue of origin. However, phenotypic drifts might be observed during prolonged cultures and obtaining a large number of primary cells is often challenging. To solve these issues, immortalized cell lines can be used. These are predominantly obtained from tumoral cells and can significantly differ from their healthy primary counterparts, leading to structural and compositional differences in the matrix they synthesize and assemble in vitro. The great advantage of employing cell lines resides in their homogeneity and stability, which also leads to an increased consistency of matrix production. Finally, variation of cell density or the use of different cell culture media can also influence matrix production and should therefore be carefully optimized and standardized for specific needs.

The process of cell culture in terms of culturing time, composition of the culturing medium, the implementation of mechanical stimulation as well as the matrix production is usually carried out in 2D environments, in which cell monolayers are maintained in culture for a prolonged time interval, or as 3D aggregates in low attachment substrates [87]. The process itself can be engineered to promote the synthesis of selected macromolecular components or to modulate their spatial assembly. For instance, ascorbic acid is usually employed to stabilize the collagen molecules and increase yielding [88,89]. Also, culturing cells in hypoxic conditions generally results in an increase of matrix production and expression of crosslinking enzymes, although the sensitivity of the biosynthetic activity to oxygen tension also depends on the cell type [90,91]. Patterned culturing substrates can be used to align cells in specific directions, thus obtaining ordered tissue structures [92,93]. Furthermore, mechanical conditioning, such as static or dynamic stretching, of cells during culture proved to improve the mechanical properties of the matrix [94,95].

To use the CDMs as cell culturing platforms for mechanobiology studies, thus exposing exogenous cells to the biochemical and the biophysical microenvironment of the CDM, a thorough decellularization is required. This creates sufficient room for exogenous cell adhesion and proliferation. However, decellularization competes with the preservation of the matrix architecture and composition. In fact, decellularization processes usually rely on harsh chemical/physical treatments that can degrade or disrupt the macromolecular components of the ECM. Combinations of acid or alkaline treatments, enzymes and surfactants are formulated to remove cell components and DNA [96,97,98]. Freeze/thawing cycles and freeze drying are effective in devitalizing the tissue, but are not apt for an efficient material removal [99]. These methods need to be carefully combined and optimized for the specific CDM in order to maximize cell removal and minimize alteration of the ECM. Chemical treatments may also remove GAGs and other small molecules, whereas physical treatments may distort the matrix structure.

Owing to the importance of the biophysical microenvironment of the tumour ECM in regulating various aspects of cancer cell behaviour including, migration, proliferation and drug resistance [28,100], CDMs represent physiologically relevant and more controllable models for fundamental research on cancer as well as for screening or testing antitumoral drugs. Several matrices derived from either tumour cells or non-tumour cells have been proposed for a variety of tumours, including oral cancer [101], breast cancer [102,103], myeloma bone disease [104], ovarian cancer and colorectal cancer [105,106]. Increased proliferation and drug resistance have been frequently reported in cases of tumour cells cultivated in CDMs with respect to cells grown on tissue culture plastic [103,107,108]. In particular, the differences in cell behaviour and response to drugs have been linked to the stiffer matrices produced by tumour or tumour-associated cells [107,109]. Also, Hoshiba and Tanaka reported increased proliferation and resistance to 5-fluorouracil of MDA-MB-231 invasive breast cancer cells on matrices produced by the same cell type, as opposed to their behaviour on matrices derived by MCF-10A benign cells [102]. These data suggest that the different ECM expression patterns associated with the different stages of tumours can be replicated in CDMs in vitro, which can be exploited to study the effects of ECM components on cancer cell behaviour or chemoresistance in specific stages of the tumour progression that are characterized by different biophysical properties.

It must be said that the use of CDM is accompanied by several technical hurdles. First, the production of matrices is time consuming and labour intensive. The biosynthesis phase may take weeks for its completion and the decellularization process requires several sequential steps that must be optimized for the specific matrix and must be carried out with great care to avoid damages to the matrix. The biosynthetic phase can be sped up with the use of inert macromolecular crowders in the culture medium that occupy volume and replicate the dense extracellular space found in the native ECM, which accelerates the collagen assembly and deposition [110]. Second, CDMs are very thin with a low stiffness in the order of 0.1–1.0 kPa [99,111,112]. Matrix staking may partly solve this problem, although the production of massive tissues could hamper the diffusion of nutrients in the bulk. Matrix stiffening can be performed with chemical agents [112,113], but this action can change the bioactivity of the support. Finally, major changes of matrix composition and structure cannot be performed in post-processing, but they must be implemented upfront by changing the cell type and/or the culturing conditions.

## 6. Decellularized Native Tissues and Organs

**Figure 4:**
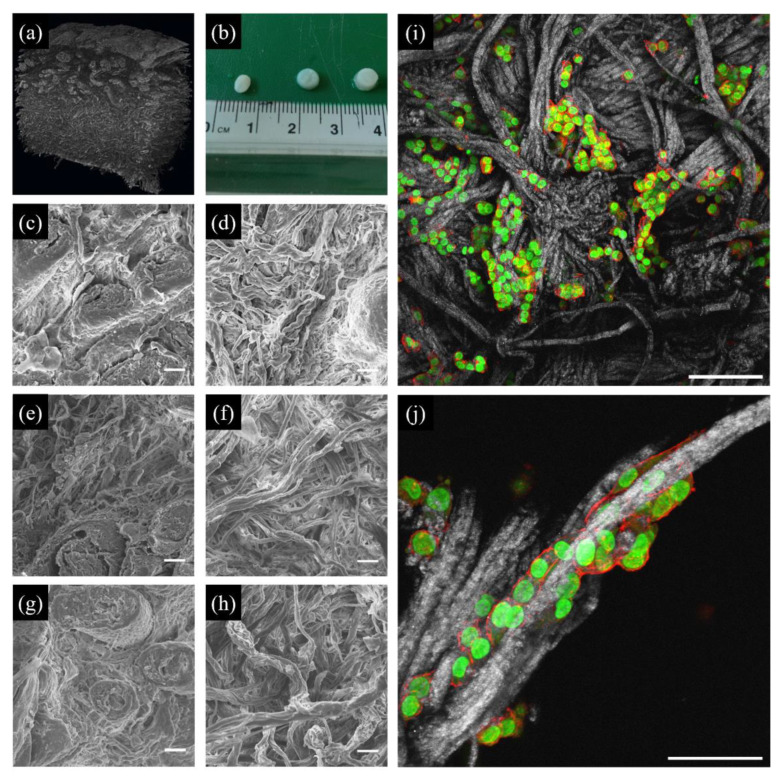
Decellularized skin substrates for cancer cell culture. (**a**) Micro-computed tomographic reconstruction showing the structure of a decellularized skin sample. (**b**) Macroscopic morphology of skin samples preconditioned at three different concentrations of phosphate-buffered saline (PBS): 1× (left), 0.1× (centre), 0.01× (right). (**c**–**h**) Scanning electron micrographs of the decellularized matrices preconditioned with 1 × PBS (**c**,**d**), 0.1 × PBS (**e**,**f**) or 0.01 × PBS (**g**,**h**). Images in c, e and g were acquired from the papillary side, d, f, and h from the reticular side. Scale bar: 20 μm. (**i**,**j**) Confocal images of A375 cells seeded on the reticular side of a decellularized skin sample collagen (grey), nuclei (green), actin (red). Bars 100 μm (**i**), 50 μm (**l**). (**a**–**h**) Reproduced with permission from Brancato et al., Journal of Tissue Engineering and Regenerative Medicine; published by John Wiley and Sons, 2017, [119]. Permissions regarding further reuse of these figures should be directed to the John Wiley and Sons.

The use of decellularized animal or human tissues may be an alternative for studying cell behaviour in a structurally more complex and mechanically competent microenvironment. Historically, decellularized tissues were conceived as replacements or augmentation agents for diseased or weakened tissues. Early examples of decellularized tissues were membrane-like tissues such as dermis and small intestine submucosa [114]. Advancements in the decellularization process led to the exploitation of highly effective methods that enable treatment of bulky tissues and organs such as the trachea, heart, liver and kidney. The use of decellularized tissues as material models for in vitro cell cultures is more recent. Owing to their intricate microarchitecture and complex composition, tuning the biochemical or biophysical features of decellularized tissues is not straightforward. Similarly to dCDMs, the stiffness of matrices derived from decellularized native tissues or organs can be modified using crosslinking chemicals including glutaraldehyde, 1-ethyl-3-(-3-dimethyl-aminopropyl)-carbodiimide, epoxy compounds, genipin and procyanidins [115]. These changes in tissue/organ mechanical properties are, however, usually accompanied by altered degradability and epitopes masking [116,117,118]. Brancato et al. proposed a simple method to alter porosity and stiffness of decellularized dermis scaffolds, which preserves degradability and fibrillar integrity through soaking the tissue in a solution containing different ionic strengths, which promotes collagen fibre repulsion and network distortion, followed by a controlled dehydration with organic solvents [119]. Samples pretreated with hypotonic solutions possessed a stiffer and more open collagen reticulum (Figure 4). Different types of cancer cells adhered and migrated through the collagen network, with the stiffer samples promoting the highest proliferation rates [119].

Several works focused on assessing the role of pathological ECM on cell behaviour in vitro. Pathologic ECMs display aberrant microstructures and altered mechanical properties compared to physiological matrices. Such changes contribute to the specification of the onset of the disease or influence its progression. For instance, idiopathic pulmonary fibrosis is a progressive disorder that can lead to respiratory failure with a prognosis of 2–5 years. No definitive therapeutic treatments are currently available, and this is partly due to the characteristics of the tissues, either in vitro-generated or isolated from animals, that scarcely reproduce the microenvironmental features that appear in the diseased tissues. Booth et al. showed that decellularized samples of human fibrotic lungs display distinctive compositional (ECM proteins and GAGs content) and biophysical (collagen and elastin fibre organization and ECM stiffness) traits compared to healthy lung matrices [120]. Furthermore, pathological matrices (i.e., matrices of pathological tissues such as fibrotic or tumoral lesions) induce a fibroblast-myofibroblast differentiation in vitro in a TGF-β-independent manner. As myofibroblasts notoriously increase matrix stiffness, this will promote phenotypic changes that can ultimately sustain or exacerbate the dynamics of the pathology [120].

Similarly, tumour stroma has been implicated not only in mechanically supporting and confining the tumour mass, but also in the progression of the pathology itself. Increased stromal stiffness is associated with drug resistance, enhanced proliferation and EMT [121,122,123]. In a recent work, Lv et al. obtained decellularized tumour matrices with different levels of LOX-mediated crosslinking [124]. MDA-MB-231 human breast cancer cells cultivated on the stiffer matrices (~2 kPa) exhibited higher resistance to the chemotherapeutic drug cisplatin compared to cells on compliant matrices (0.74 kPa). The authors reported that the increased substrate stiffness induced the overexpression of drug efflux transporters of the ATP-binding cassette family, which lowered the intracellular drug concentration. Increased levels of YAP were maintained in cells on stiff matrices, which also contributed to drug resistance and EMT [124].

These examples of dCDMs prepared from fibrotic or cancer tissues emphasize the important regulatory role of the ECM biophysical features in pathological contexts. The development and use of in vitro culturing systems such as the dCDMs provides a more controlled and accessible material platform for the investigation of complex cellular behaviour. Studies performed on histologically competent and biophysically analogous systems may potentially increase the effectiveness of drug screening experimentations, as conventional culturing platforms and animal models are distant mimics of the actual human ECM. Furthermore, as the biophysical instructions of the ECM participate in the establishment of phenotypic changes, novel therapeutics targeting the ECM may restore the correct physiologic microenvironment, possibly arresting or reverting the disease.

## 7. Future Perspectives and Conclusions

We discussed a series of culturing systems based on ECM macromolecules (chiefly collagens) aimed at replicating some of the structural, mechanical and biochemical features of the native microenvironment (Table 1). No system is actually able to capture the full complexity of the natural ECM, and these have to be intended as “reductionist” in the sense that they result from a compromise of diverse aspects. Some ECM model systems stand out for the simplicity of their fabrication, but their microstructure is way too simplified. Others require extensive processing, but result in an intricate network of fibres and bundles very similar to natural tissues. Of course, the suitability of a model depends on its final application. 2D coatings and dCDMs are particularly suitable to study cell adhesion and migration processes, as they are directly accessible and cells can be seeded by sedimentation. They are also sufficiently thin to be visualized with high magnification lenses. Whereas 2D coatings can be implemented in a relatively straightforward manner, dCDM processing requires much longer times, as matrix deposition usually takes weeks, and the subsequent decellularization is very labour-intensive. Also, the production of CDMs may not allow an easy and immediate modification of the architecture and stiffness of the tissue. Changing these properties requires the adoption of specific strategies in pre- or post-processing.

With respect to 2D substrates and dCDMs, 3D fibrillar gels are much more versatile systems as they are easy to manufacture, their transparency enables direct visualization and the porous network makes them ideal platforms to study cell migration in the presence of chemotactic factors. However, their stiffness is low if compared to native ECMs. Most importantly, biomolecular gels do not allow achievement of an orthogonal control over morphology, mechanical properties and composition, i.e., any change in one parameter also affects the others. Finally, decellularized tissues and organs are the systems that more closely replicate the native counterparts in terms of structure and composition. However, proper decellularization is a very elaborate and time-consuming procedure. Furthermore, differently from the other systems, re-cellularization may present significant technical hurdles, especially for ensuring a homogeneous cell distribution in bulky tissues. Under these circumstances, perfusion reseeding is usually performed, which requires the use of specialized bioreactors. These are also needed to sustain the culture itself as the thick ECM limits nutrient diffusion.

To overcome this issue, several types of tissue/organ extracts or homogenates have been proposed. Some of these retain the ability to self-assemble and gelify under mild physiologic conditions enabling direct cell encapsulation at the expense of the microarchitecture that is disrupted. These gels possess a fine fibrillar network constituted by a variety of microconstituents including collagens (fibril forming and non-fibrillar), fibronectin, GAGs and laminins, whose amounts strongly depend on the tissue of origin [125,126]. This is a great advantage compared to single component gels such as collagen or fibrin gels. However, being the precursors extracted from natural tissue, their composition may show some differences from batch to batch and, also in this case, the mechanical properties of the reconstituted gels are considerably low [127]. Despite the lack of a well-defined structure and weak mechanical stability, tissue-derived gels find interesting applications as bioinks for 3D printing applications. Tissue-derived gels have been used alone or in combination with synthetic materials (for example, Polycaprolactone) for mechanical support [128,129].

Although tissue-derived gels provide some level of flexibility and versatility in engineering and fabricating systems for in vitro cell cultures, today a technology able to exactly reproduce ECM analogues with native biochemical/biophysical features does not exist. Such a technology should handle a myriad of very labile biomolecules and deliver them in space with a nanoscale precision, and we do not expect that this would be feasible in the near future. Even if we had this powerful technology at our disposal, still we would face other issues. First, as cells and ECMs mutually affect each other and together define the functions of the tissue itself, matrices deprived of their cellular component might not provide the same mechanically instructive environment. Second, whatever culturing system is used, cells secrete macromolecules that invariably alter the chemical-physical properties of the microenvironment. This aspect must be taken into account when drawing out conclusions on the cell response to extracellular stimuli, especially in cases of prolonged cultures.

With this in mind, a very close approximation of a native tissue or organ is a biological system constituted by cells (or different cell types) and an endogenously produced ECM. Along these lines, Netti and co-workers exploited a bottom-up approach, in which microtissues grown on degradable carriers self-assemble in maturation chambers to generate viable and histologically competent tissues [130,131]. With this technique, they were able to produce libraries of organotypic tissues including innervated or vascularized skin [132], tumour tissues and the associated stroma [133], uterine cervix [134] and intestine [135]. These models displayed superior mechanical properties (static and dynamic) with respect to matrices made from exogenous materials, a heterogeneous composition sharing similar spatial patterns as the native one and, most importantly, a microarchitecture in which the microconstituents are arranged in supramolecular structures such as those observed in vivo. Thanks to these analogies, bottom-up processed tissues display very peculiar biological properties. For instance, microtissues better recapitulate the morpho-mechanical and biological differences that are observed between normal and cancer-activated stroma. Also, cervical tumour models composed of cervical cancer-associated fibroblasts and cervical cancer epithelial cells provide superior environments to mimic cervical carcinogenesis in vitro as opposed to conventional rafts models. The bottom-up assembly process is particularly suitable to culture microfluidic devices (mFLds), in which microtissues may directly self-assemble into the device, thus generating proper organotypic tissue-on-chips or organ-on-chips systems [136,137], which is a great advantage compared to conventional cell monolayers grown in mFLds. Despite these enormous benefits, the technology is time consuming as tissue maturation may take weeks, and also suffers the same limitations of tissue- and organ-derived matrices, i.e., the mechanical, biochemical and structural features cannot be readily manipulated in post-processing.

Clearly, several biomimetic systems for mechanobiology studies are currently available, each capturing one or more features of the native environment, and are therefore suitable for specific applications. A question, however, remains unanswered: do we have robust analytical tools to assess how similar a biomimetic system is to native tissues or organs? According to an old quote, “The best material model for a cat is another, or preferably the same cat”. In the context of mechanobiology, the research of the “best material model” is hindered by technological issues and, most importantly, by our limited understanding of the involvement of mechanobiology in health and disease. Rather than seeking to exactly replicate any possible biochemical/biophysical feature on a biological system, it would be more appropriate and efficient to validate key variables (i.e., local density, stiffness, porosity and architecture) and establish logical relationships among them. This requires possessing a certain level of ab initio knowledge of the system of interest. We envision that integrating high throughput molecular analyses, such as RNA-seq, high resolution intravital microscopy approaches, and in situ mechanical characterization techniques, such as multiparticle tracking or ultrasonic microelastography, would provide sufficient elements to identify the most relevant biochemical/biophysical features involved in in vivo mechanotransduction. Mathematical modelling can be a valid instrument in understanding the complex dynamics of the molecular machinery of cell force generation and transmission. Molecular clutch models have been successfully applied to describe actin and cell migration dynamics as well as to interpret mechanotransduction events in in 1D or in 3D contexts [138,139]. Indeed, some models incorporate force-dependent adhesion reinforcement that better captures the cell-generated force–ECM stiffness relationship [140,141]. Also, substrate viscoelasticity and stored strain energy have been added, thus enhancing the predictive capabilities and versatility of the models [142,143,144]. Inside-out force transmission is crucial in the matrix remodelling, for which computational models can enable the prediction of the spatiotemporal changes of the ECM microarchitecture and mechanics that can feedback time evolving mechanical stimuli to cells. Network-based models provide a deeper insight into the defamation mode (i.e., bending or stretching) of the fibrillar matrix around contractile cells according to their shape [145], as well as the long-range effect of the cell-generated deformation fields that allow cells to sense each other at large distances [146].

Altogether, these improvements would foster the rational re-engineering of the ECM for targeted in vitro studies. By combining material models replicating the relevant features with sensing/actuating devices such as mFLds or microelectromechanical systems, devices would allow mechanical stimulation and acquire cell responses in a highly controlled and much prompter manner. The rational design of material models is only one part of a wider problem involving different analytical and controlling techniques that need to be carefully addressed in order to obtain reliable and consistent data for an effective clinical translation of the findings. As biomimetics continue to grow in complexity and versatility, so do biomathematical models [147]. In the future, we expect this powerful combination, together with emerging sophisticated machine learning algorithms [148], to aid in developing innovative diagnostic routes based on altered matrix structures or mechanical properties and in designing novel therapies aimed at targeting the biophysical and mechanical features of the tumour ECM to increase therapy efficacy.

## Figures and Tables

**Table 1:** Summary of the culturing models presented in the text reporting a schematic representation of the systems, their biophysical properties, advantages, disadvantages and their use in cancer studies.

	2D Fibrillar Collagen Substrates 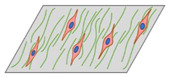	3D Collagen Gels 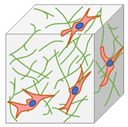	dCDMs 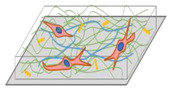	Decell. Tissues 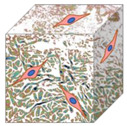
Tuneable biophysical property	Collagen surface concentration, periodicity, fibril strength, orientation	Collagen density, porosity, stiffness, fibril length	Fibril density, stiffness	Matrix stiffness, porosity
Advantages	Ease of fabrication; directly accessible to optical, fluorescence, confocal and AFM microscopy or mechanical probing	Accessible to optical, fluorescence, confocal microscopy. Homogeneous cell encapsulation	Biomimetic fibrillar environment; accessible to optical, fluorescence, confocal and AFM microscopy or mechanical probing	Tissue-like environment
Limitations	Mostly grown on mica; not directly applicable on hydrogels or elastomers	Independent control of the biophysical parameters is not straightforward; mechanical and microstructural features are generally dissimilar to native tissues	Limited control of the manipulation of the biophysical parameter; tissue growth and decellularization are time consuming; limited manipulations in post processing	Heterogeneous cell distribution; limited control of the manipulation of the biophysical parameter; decellularization is time consuming; limited manipulations in post processing; impaired nutrient transport for bulky tissues
Use in cancer studies	MV3 cell morphology; collagen remodelling; MMP expression [47]; MDA-MB-231 and MTLn3 morphology, adhesion, migration [48]; MDA-MB-231 and MTLn3 cell adhesion and motility [49]; SKOV3 cell morphology, migration, cell mechanics, gene expression [50]	HMT3522 and MCF10A MEC cell adhesion, morphology, colony formation, gene expression, protein localization, cell contractility [60]; MDA-MB-231 cell morphology, migration [64]; MDA-MB-231 cell morphology, migration, response to drugs [73]	MDA-MB-231, MCF-7 and MCF-10A cell adhesion, proliferation, gene expression [102]; MDA-MB-231 cell metabolism, proinflammatory profile, adhesion protein translation, MMP activity, drug response [103]; MDA-MB-231, primary SCC and CAF cell proliferation, gene expression, protein translation, tumour growth [107]; breast, colorectal, lung, pancreatic, ovarian cell proliferation, morphology, drug response [108]	MCF-7, PT45 and A375 cell adhesion, proliferation [119]; MDA-MB-231 cell proliferation, infiltration, apoptosis, drug response [124]
